# Performance of a high sensitivity time-of-flight PET ring operating simultaneously within a 3T MR system

**DOI:** 10.1186/2197-7364-1-S1-A72

**Published:** 2014-07-29

**Authors:** Craig S Levin, Floris Jansen, Tim Deller, Sri Harsha Maramraju, Alex Grant, Andrei Iagaru

**Affiliations:** Stanford University, Kragujevac, USA; GE Healthcare, Kragujevac, USA

A time-of-flight (TOF)-PET/MR research system installed at Stanford will be used to test the hypotheses that (a) it is possible to acquire simultaneous TOF-PET and 3T MR data while achieving uncompromised performance in both modalities and (b) simultaneous TOF-PET/MR is a tool for multi-parameter characterization of disease. In this paper we will describe the design as well as performance measurements both for the standalone PET ring, and with the two systems integrated. We will also show a selection of clinical images to compare the performance of the integrated TOF-PET/MR system with that of a state-of-the-art PET/CT system.

The silicon photomultiplier (SiPM)-based TOF PET system technology under study was integrated into a modified wide-bore 3T MR scanner. Measurements were performed using the full suite of NEMA NU2-2007 protocols; these protocols were executed with MR on and off to evaluate the influence of MR on the performance of PET. The diagnostic imaging performance was evaluated using subjects who had been already injected with ^18^F FDG for a clinically indicated PET/CT exam.

The high photon sensitivity and high temporal, energy, and spatial resolutions observed are minimally affected by the concurrent MRI acquisition. Imaging of the first patients showed: (1) High quality images with very low background were achieved, due to the combination of high photon sensitivity, TOF, and long delay post-injection; (2) The combination of high photon sensitivity and high spatial resolution enabled enhanced visualization of small anatomical and abnormal structures; (3) Quantitation of areas of abnormal uptake showed statistically significant increase in SUV_max_ as expected after the additional uptake time

The data indicate that the TOF PET system is capable of excellent performance during simultaneous PET/MR. Comparison of the PET/MR and PET/CT images show equivalent or better quality of the PET images for the PET/MR system.

